# Engineering a humanized telomerase reverse transcriptase gene in mouse embryonic stem cells

**DOI:** 10.1038/s41598-019-46160-5

**Published:** 2019-07-04

**Authors:** De Cheng, Yuanjun Zhao, Fan Zhang, Jinglong Zhang, Shuwen Wang, Jiyue Zhu

**Affiliations:** 1 Department of Pharmaceutical Sciences, Washington State University College of Pharmacy and Pharmaceutical Sciences, Spokane, Washington, USA; 2 Department of C & M Physiology, Pennsylvania State University College of Medicine, Hershey, Pennsylvania USA

**Keywords:** Cancer models, Telomeres

## Abstract

Telomerase is expressed in adult mouse, but not in most human, tissues and mouse telomeres are much longer than those in humans. This interspecies difference of telomere homeostasis poses a challenge in modeling human diseases using laboratory mice. Using chromatinized bacterial artificial chromosome reporters, we discovered that the 5′ intergenic region, introns 2 and 6 of human telomerase gene (*hTERT*) were critical for regulating its promoter in somatic cells. Accordingly, we engineered a humanized gene, *hmTert*, by knocking-in a 47-kilobase hybrid fragment containing these human non-coding sequences into the *mTert* locus in mouse embryonic stem cells (mESCs). The *hmTert* gene, encoding the wildtype mTert protein, was fully functional, as a mESC line with homozygous *hmTert* alleles proliferated for over 400 population doublings without exhibiting chromosomal abnormalities. Like human ESCs, the engineered mESCs contained high telomerase activity, which was repressed upon their differentiation into fibroblast-like cells in a histone deacetylase-dependent manner. Fibroblast-like cells differentiated from these mESCs contained little telomerase activity. Thus, telomerase in mESCs with the *hmTert* alleles was subjected to human-like regulation. Our study revealed a novel approach to engineer a humanized telomerase gene in mice, achieving a milestone in creating a mouse model with humanized telomere homeostasis.

## Introduction

Telomeres are specialized nucleoprotein structures at chromosomal ends and play key roles in human aging and tumorigenesis. Telomeric DNAs are replenished by telomerase, a ribonucleoprotein complex containing two essential components, a catalytic reverse transcriptase (TERT) and a template RNA (TERC), along with accessory proteins^1,2^. In humans, telomerase activity is low or undetectable in most adult tissues. Consequently, telomeres shorten upon successive cell divisions, thereby functioning as a biological clock. In contrast, telomerase activity is readily detected in adult mouse tissues and laboratory mice have very long telomeres (50–100 kb)^3^. Consequently, telomerase-null mice survive up to six generations with no discernible phenotypes in early generations^4–6^.

Studies have indicated that hTERT transcription is the primary step of telomerase regulation^7^. Whereas TERC RNA and telomerase-associated proteins are expressed in most somatic tissues, hTERT expression is highly regulated and correlated with telomerase activity in many adult cell types^8^. hTERT transcriptional regulation is a complex process that involves both the binding of transcription factors at its promoter and epigenetic repression that regulate chromatin environment of the *hTERT* locus^9^. Our previous studies revealed that the *hTERT* gene was embedded in a condensed chromatin domain in somatic cells and inhibition of histone deacetylation led to an opening of this domain and activated hTERT transcription^10^. We have recently shown that repression of the hTERT promoter was dictated by distal elements and required a human specific genomic context^11,12^.

The differences in telomerase regulation and telomere length among different species likely contribute to important differences in aging processes and cancer development between humans and mice. Indeed, many human somatic cells, but not mouse cells, undergo replicative senescence mediated by progressive telomere shortening^13^. Mouse models often give rise to tumors of different spectra from the types of human cancers they are intended to mimic. For example, p53-null mice of several inbred strains typically developed soft tissue sarcomas and lymphomas, the types of cancer mostly occurring in human childhood^14^. Conversely, p53 deficiency, along with shortened telomeres in late generations of telomerase-null mice resulted a shift of tumor spectrum to epithelial carcinomas of skin, breast, and colon origins, the most common adult human cancers^15^. Thus, telomere length and telomerase regulation contribute to the different tumor development in mouse and human cancers. Nonetheless, telomerase-null mice are not the best models for human cancers because their telomerase cannot be reactivated as it occurs in majority of human cancers. An ideal model would regulate telomerase in mice the same way as in humans. Here, as the first step towards the generation of a mouse model with humanized telomerase regulation and telomere homeostasis, we determined the *cis* regulatory elements involved in hTERT repression and revealed that human genomic sequences within the 5′ intergenic region (5IR), introns 2 and 6 (I2 and I6) contained elements that confer a human-like regulation in a mouse genomic context in telomerase-positive and telomerase-negative fibroblast lines. These elements also repressed the hTERT promoter during mouse ESC differentiation. We thus engineered an *mTert* locus by replacing its 5IR, I2, and I6 with corresponding human genomic sequences in mouse ESCs. Our results showed that mouse ESCs with the chimeric locus, *hmTert*, expressed a high level of telomerase activity and this activity was strongly repressed during *in vitro* differentiation, similar to human ESCs.

## Results

### Regulatory genomic sequences required for hTERT repression

**Figure 1:**
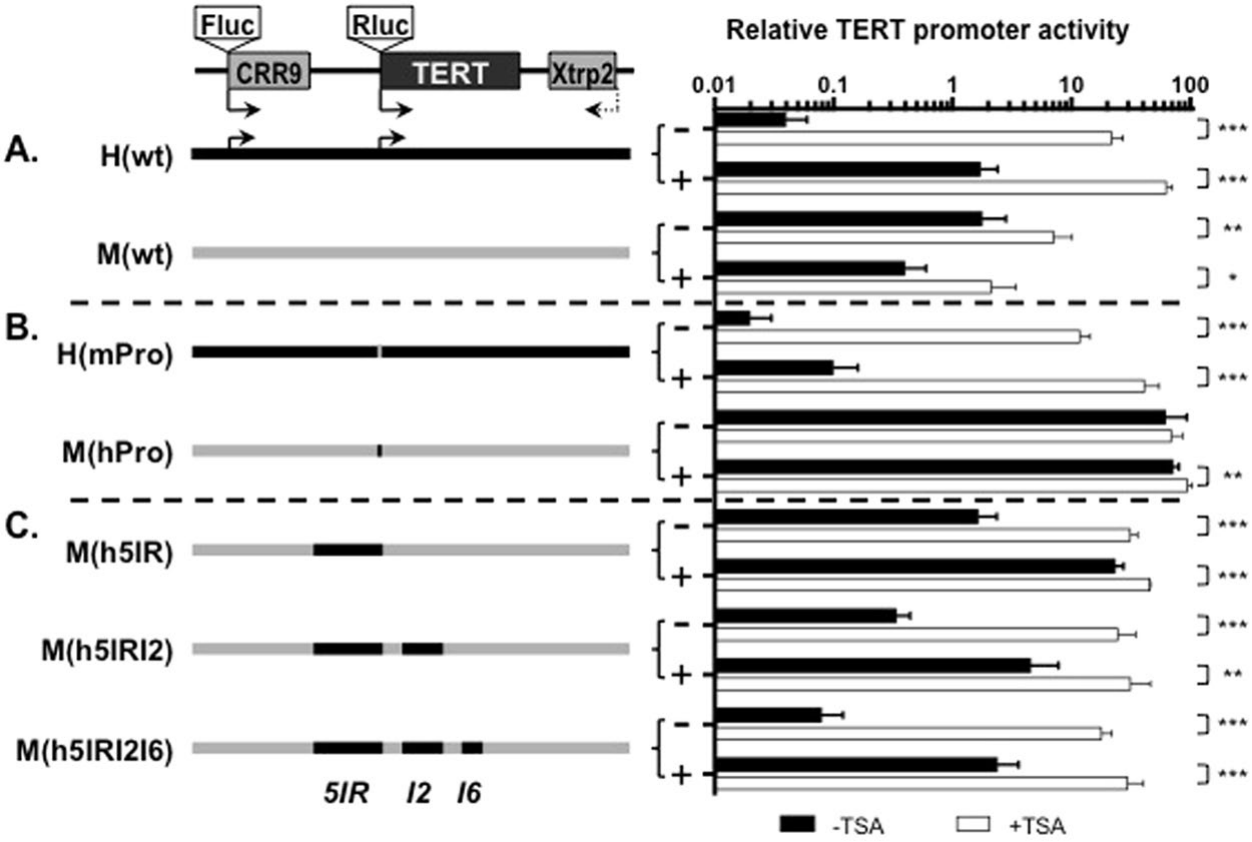
The involvement of distal genomic sequences in the repression of hTERT promoter in human fibroblasts. A schematic diagram of all BAC reporters is shown at the top. Shown below are illustrations of H(wt), M(wt), and chimera BACs. Black and light grey lines represent human and mouse genomic sequences, respectively. (**A**) BAC reporters made from human and mouse genomic sequences, H(wt) and M(wt), respectively. (**B**) H(mPro) and M(hPro), in which *TERT* promoter regions were swapped between H(wt) and M(wt). (**C**) M(h5IR), M(h5IRI2) and M(h5IRI2I6), *5IR*, *I2*, and *I6* represent 5′ intergenic region, introns 2 and 6 of the *hTERT* gene, replacing their corresponding mouse sequences in M(hPro). Bar graphs, activities of TERT promoters in chromatinized BAC reporters in Tel^−^ (−) and Tel^+^ (+) human fibroblast cells. Luciferase activities were measured in 96-well plates, treated without (black bars) or with TSA (white bars). The *TERT* promoter activities were measured as the ratios of *Firefly* to *Renilla* luciferase activities. *p < 0.05; **p < 0.01; ***p < 0.001.

Histone deacetylase (HDAC)-dependent transcriptional repression is central to the regulation of *hTERT* gene in somatic cells^16^. To identify the genomic sequences responsible for the differential regulation of *hTERT* and *mTert* genes, we used the BAC reporters H(wt) and M(wt)^17^, which contained a 160-kb and a 135-kb genomic DNA encompassing the consecutive *CRR9* (also called *CLPTMIL*), *TERT*, and *XTRP2* (*SLC6a18*) loci of human and mouse genomic sequences, respectively (Fig. 1). Single-copy BACs were integrated into the same acceptor sites in Tel^+^ and Tel^−^ human fibroblast lines by recombinase-mediated BAC targeting (RMBT)^17^, and thus had the same genomic environment. In this system, the activities of TERT promoters were measured as the ratios of *Renilla* luciferase *(Rluc)* activities from *TERT* promoters to Firefly luciferase (*Fluc*) activity from CRR9 promoters, because CRR9 promoters were constitutively active in most cells^17^. As shown in Fig. 1A, the hTERT promoter in H(wt) was 30–50 fold more active in Tel^+^ cells than that in Tel^−^ cells and markedly activated in both cells by HDAC inhibitor trichostatin A (TSA), recapitulating the endogenous expression of *hTERT* gene^17^. While the mechanism of hTERT activation in Tel^+^ versus Tel^−^ cells remained to be elucidated, it is likely that Tel^+^ cells contained different *trans*-acting factors, either increased activators or reduced repressors, that interact with the hTERT *cis*-regulatory elements. On the other hand, the mTERT promoter in M(wt) was moderately active in both Tel^+^ and Tel^−^ cells. In our recent report^12^, the hTERT and mTert promoters, which were 472-bp and 474-bp genomic sequences upstream of the hTERT and mTert ATG codons, respectively, were swapped between H(wt) and M(wt), generating H(mPro) and M(hPro). Upon integration into Tel^+^ and Tel^−^ cells by RMBT. The mTert promoter activity in the human genomic context, H(mPro), was higher in Tel^+^ cells than in Tel^−^ cells and strongly activated by TSA in both cells (Fig. 1B). In contrast, the hTERT promoter in the mouse genomic context, M(hPro), was highly active in both Tel^+^ and Tel^−^ cells, and TSA treatment had little or no effect on its activity. Thus, the human, but not the mouse, genomic sequence conferred a repressive chromatin environment for both TERT promoters^12^.

Whereas the coding regions are conserved between *hTERT* and *mTert* loci, non-coding regions are highly diverged both in sequences and in lengths. For example, 5IR of the *hTERT* gene is over 22 kb, yet its mouse counterpart is about 6 kb. The two largest introns, I2 and I6, are 10 kb and 5.5 kb in human, and 5.5 kb and 1.8 kb in mice, respectively. To determine if these regions are important for human-specific repression, we replaced the regions in M(hPro) with their human counterparts, generating M(h5IR), M(h5IRI2), and M(h5IRI2I6). As shown in Fig. 1C, 5IR replacement in M(hPro), i.e. M(h5IR), resulted in a 2-fold and a 20-fold HDAC-dependent repression of the hTERT promoter in Tel^+^ and Tel^−^ cells, respectively. Inclusion of human I2 and I6, i.e. M(h5IRI2) and M(h5IRI2I6), led to more stringent repression of the hTERT promoter in these cells: 7 fold in Tel^+^ and 73 fold in Tel^−^ cells for M(h5IRI2), and 15 fold in Tel^+^ and over 200 fold in Tel^−^ cells for M(h5IRI2I6). Accordingly, the hTERT promoter in M(h5IRI2I6) was about 30 fold stronger in Tel^+^ cells than that in Tel^−^ cells, similar to the endogenous promoter in host cells^17^. Therefore, all three non-coding regions of the *hTERT* gene contributed to an hTERT-like regulation in the context of chromatinized BAC reporters in human fibroblasts.

### Regulatory functions of hTERT non-coding regions during ESC differentiation

**Figure 2:**
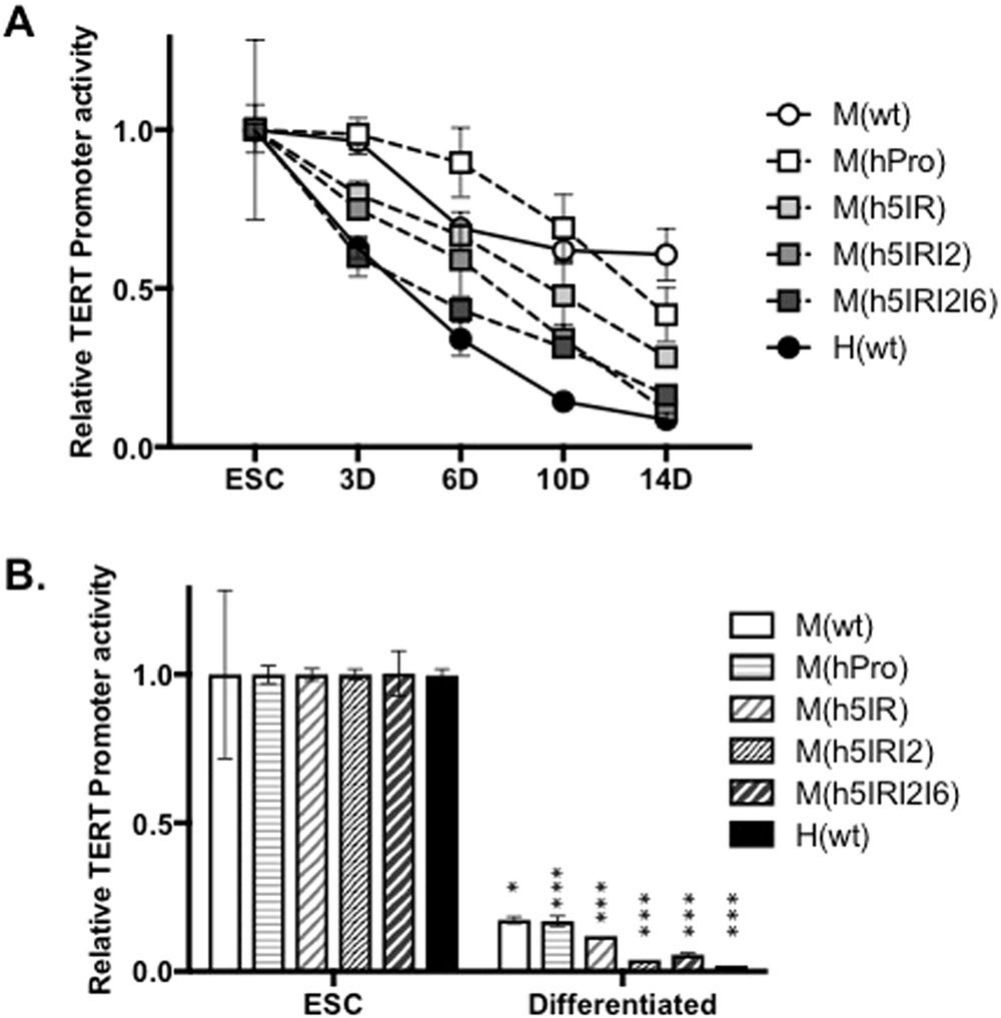
TERT promoter activities from chromatinized BAC reporter in mouse ESCs and their differentiated derivatives. (**A)** TERT promoter activities in differentiating EB cultures. ESCs containing BAC reporters were cultured in the absence of feeder cells and LIF to form EBs. Luciferase activities were measured in 3, 6, 10, and 14 days, and normalized to those of the undifferentiated ESCs. (**B**) TERT promoter activities in differentiated fibroblast-like cultures. The promoter activities were normalized to those of ESCs. *p < 0.05; ***p < 0.001, compared to their respective activities in ESCs.

Next, we determined whether the human genomic sequences conferred human-like TERT regulation in mouse cells. A subset of aforementioned BAC reporters were integrated into the same acceptor site in a mouse ESC line T2-5 by RMBT^11^. The TERT promoters in all the BAC reporters, M(wt), M(hPro), M(h5IR), M(h5IRI2), and M(h5IRI2I6), were highly active in ESCs as determined by ratios of *Rluc*/*Fluc*. To determine the regulation of TERT promoters in these chimera BACs during *in vitro* differentiation, ESCs were induced to differentiate in suspension cultures in the absence of leukemia inhibitory factor (LIF). Upon differentiation into embryoid bodies (EBs) for two weeks, the mTert and hTERT promoters in M(wt) and M(hPro) decreased by 40% and 55%, respectively, compared to their levels in undifferentiated ESCs (Fig. 2A). Addition of the 5IR in M(h5IR) led to a 70% decrease of hTERT promoter activity. In M(h5IRI2) and M(h5IRI2I6), the hTERT promoter was reduced by 85–90%, comparable to 90% of H(wt). These results indicated that human 5IR, I2, and I6 functioned to regulate hTERT promoter during mouse EB differentiation.

Although EBs contained a variety of differentiated cells, there were also undifferentiated and partially differentiated cells with significant hTERT promoter activities in these structures. To study the repression of the hTERT promoter in differentiated cells, adherent cells from EBs were enriched by removing detached cells and cultured in osteogenic medium for an additional three weeks to allow their differentiation into fibroblast-like cells. During this process, the mTert and hTERT promoters in M(wt) and M(hPro) decreased to 16–18% of their activities in ESCs (Fig. 2B); the hTERT promoter was reduced to 12% in M(h5IR), and 4–6% in M(h5IRI2) and M(h5IRI2I6), compared to their respective activities in ESCs. The hTERT promoter activities in M(h5IRI2) and M(h5IRI2I6) was slightly higher than that of H(wt) at 2%. Thus, inclusion of the three non-coding regions of the *hTERT* locus, 5IR, I2 and I6, resulted in hTERT-like regulation in an otherwise mouse genomic context during mouse ESC differentiation.

### Engineering a humanized mTERT locus (hmTERT)

**Figure 3:**
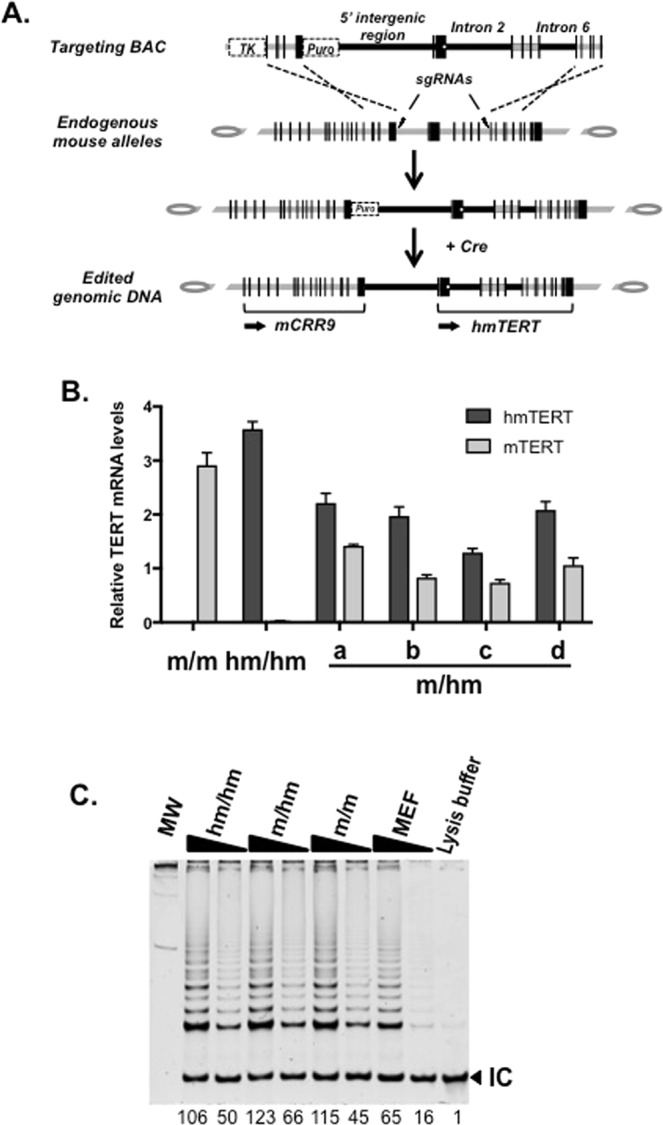
Engineering of an *hmTert* allele in mouse ESCs. (**A**) A diagram of the targeting BAC. Black and grey portions of horizontal lines indicate human and mouse genomic sequences, respectively. Vertical bars show relative positions and sizes of exons of the CRR9 (left) and TERT (right) genes. TK and Puro are thymidine kinase and puromycin-resistant markers, respectively. The targeting BAC along with two pX458-sgRNAs targeting mouse sequences near the boundaries of homologous recombination (indicated by flash signs) were cotransfected into ESCs and puromycin/GCV-resistant colonies were selected. The Puro marker was subsequently removed following transient transfection of pCBM, a Cre-expressing plasmid. The knock-in of a 47-kb genomic region as the result of homologous recombination is indicated. A small white dot at the 3′ end of Tert exon 2 indicates the silent mutations that distinguish mRNAs from *mTert* and *hmTert* loci in RT-qPCR assays. (**B**) mRNA expression from the *hmTert* locus. Total RNAs from ESCs containing *mTert* and/or *hmTert* loci were subjected to RT-qPCR analysis using probes that distinguish mTert and hmTert mRNAs, and the data were normalized to 18S rRNA. a-d were four independent m/hm clones. (**C**) Telomerase activity in ESCs with *mTert* and/or *hmTert* alleles. Telomerase activities in extracts of 2000 or 20 cells were measured using TRAP assay. m/m, wildtype ESCs containing two *mTert* alleles; hm/hm, ESCs containing *hmTert*/*hmTert* alleles, m/hm, heterozygous ESCs with *mTert*/*hmTert* genes. MEF, wildtype mouse embryonic fibroblast cells. RNase A treated wildtype ESC extract was used as a negative control. MW, molecular weight control. IC, internal control. Underneath are relative intensities of all telomere bands in each lane, normalized to lysis buffer control (1).

To determine whether the non-coding genomic sequences confer human-like regulation at the endogenous *mTert* gene, we set out to engineer a humanized *mTert* gene, or *hmTert*, by knocking human 5IR, I2, and I6 into the *mTert* locus in ESCs via homologous recombination. Hence, a chimeric targeting BAC was constructed as illustrated in Fig. 3A. This construct contained a 47-kb hybrid region containing the human 5IR, I2, and I6, replacing the corresponding mouse sequences. The mTERT protein coding sequence was preserved except for the 9 silent nucleotide substitutions near the 3′ end of exon 2, allowing the design of PCR primers that distinguished mRNAs from the *hmTert* and wildtype *mTert* alleles. The targeting BAC was transfected into Tc1 ESCs together with two plasmids expressing sgRNAs targeting mouse 5IR and I6. The cells were selected sequentially for resistance to puromycin and ganciclovir (GCV), and the resulting clones were analyzed for the presence of the entire 47-kb genomic region resulting from homologous recombination by genomic PCR and Southern analyses as shown in Fig. S1A,B. From 800 puromycin-resistant clones, we identified one ESC clone that contained two *hmTert* alleles (*hm*/*hm*) and multiple clones with a single *hmTert* allele (*m*/*hm*) (Table S1). To avoid the potential effects of off target cleavage by Crispr/Cas9, we have analyzed multiple independent clones in subsequent studies.

### The hmTERT gene maintained telomeres in ESCs

**Figure 4:**
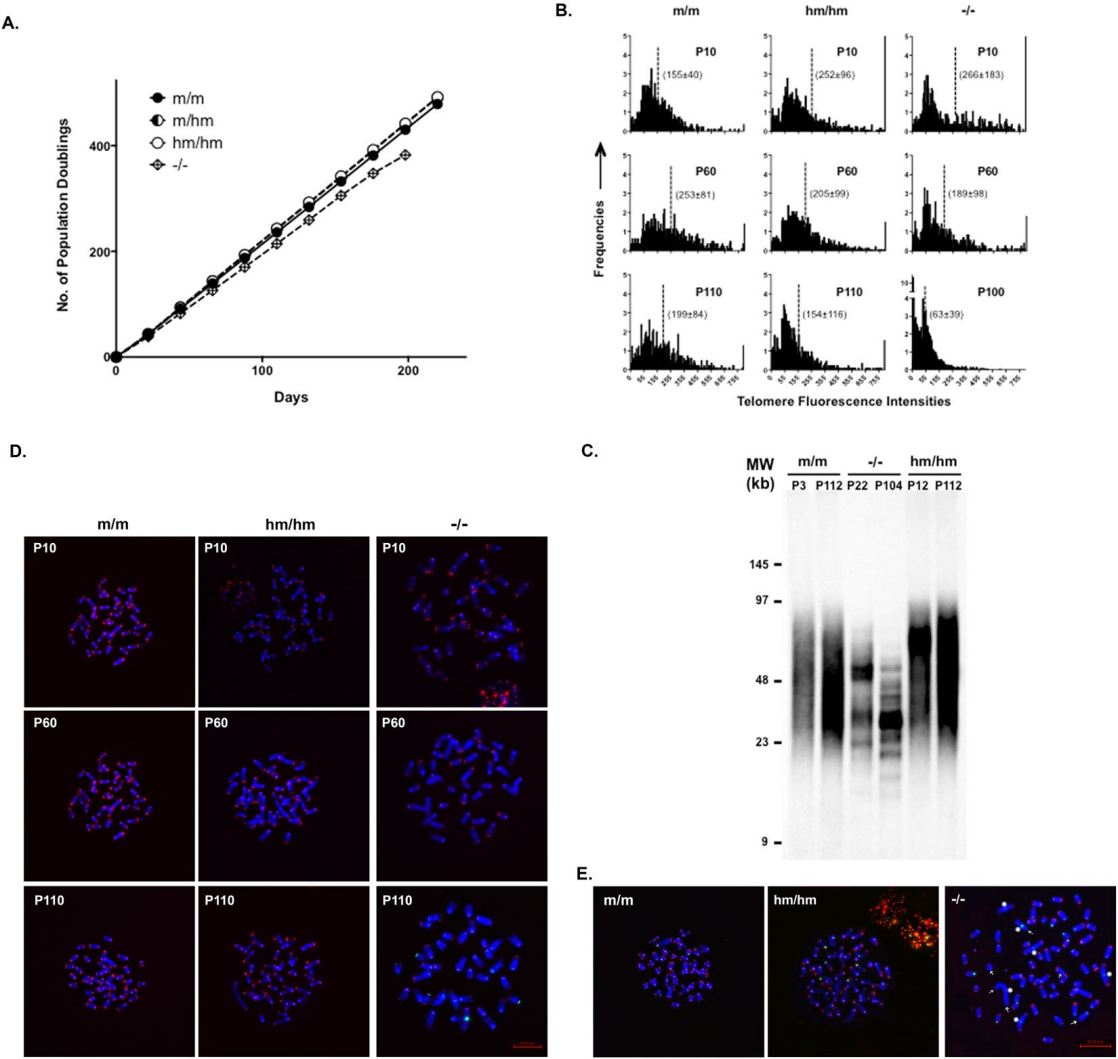
Proliferation and chromosome stabilities of ESCs with *mTert* and *hmTert* alleles. (**A**) Long-term proliferation of ESCs. ESCs with *mTert*/*mTert* (*m*/*m*), *mTert*/*hmTert* (*m*/*hm*), *hmTert*/*hmTert* (*hm*/*hm*), or mTert homozygous knockout (−/−) were passaged every 2 days for 110 times (*m*/*m*, *m*/*hm*, & *hm*/*hm*) or 100 times (−/−). (**B**) Telomere length in ESCs. Telomere lengths (Cy3) were measured by quantitative FISH (qFISH) analysis and the data were analyzed using the Metasystems software package. Mean telomere lengths are indicated by vertical dash lines and numbers in parentheses (mean ± s.d.). (**C**) Telomere restriction fragment length analysis. Genomic DNAs were extracted from ESCs at various passages and digested with HinfI and RsaI, followed by pulsed-field gel electrophoresis and Southern blotting. MW, molecular weight. Genotypes and passage numbers are indicated above each lane. (**D**) Images of telomeres (Cy3) in ESCs. (**E**) Telomere and chromosome integrity in ESCs. Red, Cy3 for telomere; green, FAM for centromere; blue, DAPI for DNA. Arrows point to telomere signal-less chromosomal ends and stars indicate chromosomal end-end fusion.

To determine the functionality of *hmTert* gene, ESC clones with *hmTert* alleles were further characterized. The *hm*/*hm* clone and two *m*/*hm* clones proliferated similarly to the wildtype *m*/*m* ESCs and had a correct number of 40 chromosomes (Fig. S2). The levels of Tert mRNA in a wildtype (*m*/*m*), an *hm*/*hm*, and four *m*/*hm* clones were determined by RT-qPCR analysis (Fig. 3B). Tert mRNA from the *hmTert* alleles was moderately higher (up to 2-fold) than those of the *mTert* alleles in both *hm*/*hm* and *m*/*hm* ESCs. Telomerase activities in *hm*/*hm* and *m*/*hm* ESCs were also similar to that in wildtype ESCs (*m*/*m*), as measured in a semi-quantitative TRAP assay (Fig. 3C). To assess the function of *hmTert* locus, ESCs were passaged for over 100 times (400 population doublings). Wildtype (*m*/*m*), heterozygous (*m*/*hm*), and homozygous (*hm*/*hm*) ESCs all proliferated similarly, whereas the proliferation of an ESC clone with homozygous *mTert* deletion (*mTert* −/−) slowed down significantly in late passages (Fig. 4A). The lengths of telomere in ESCs at different passages were measured by telomere fluorescence *in situ* hybridization (FISH) analysis and telomere restriction fragment analysis. Whereas telomere length varied during passages in both *m*/*m* and *hm*/*hm* clones, telomeres became significantly shorter in *mTert* −/− cells after 100 passages (Fig. 4B,C). Only 0.3 telomere signal-free chromosomal ends per metaphase were found in *hm*/*hm* cells after 110 passages, similar to *m*/*m* cells (Fig. 4D and Table 1). In contrast, about ten signal-free ends per metaphase were detected in *mTert* −/− cells at passage 100. Finally, while an average of five chromosomal end-end fusions were detected in *mTert* −/− cells after 100 passages, no such fusion was detected in *hm*/*hm* cells (Fig. 4E and Table 1). Taken together, these data indicated that *hmTert* loci encoded a functional telomerase gene and were able to maintain telomere stability in long-term cultures of mouse ESCs.

**Table 1:** Telomere FISH analysis of chromosomal abnormalities in ESCs.

ESC genotypes	Passage numbers	# of metaphases	# of chromosomes	# of signal-free ends (per metaphase)	# of end-to-end fusions (per metaphase)
*m*/*m*	10	15	599	0/5(0)	0/15 (0)
*m*/*m*	60	15	602	1/18 (0.06)	0/15 (0)
*m*/*m*	110	18	726	5/18 (0.28)	1/18 (0.06)
*hm*/*hm*	10	19	734	1/19 (0.05)	0/19 (0)
*hm*/*hm*	60	15	609	1/15 (0.07)	0/15 (0)
*hm*/*hm*	110	15	597	5/15 (0.33)	0/15 (0)
−/−	10	16	678	10/17 (0.59)	1/16 (0.06)
−/−	60	15	604	74/15 (4.93)	14/15 (0.93)
−/−	100	17	739	172/17 (10.12)	94/17 (5.53)

Note: Most of chromosomal end fusion and signal-free occurred on the short arm of chromosomes

### The hmTERT loci were repressed during ESC differentiation

**Figure 5:**
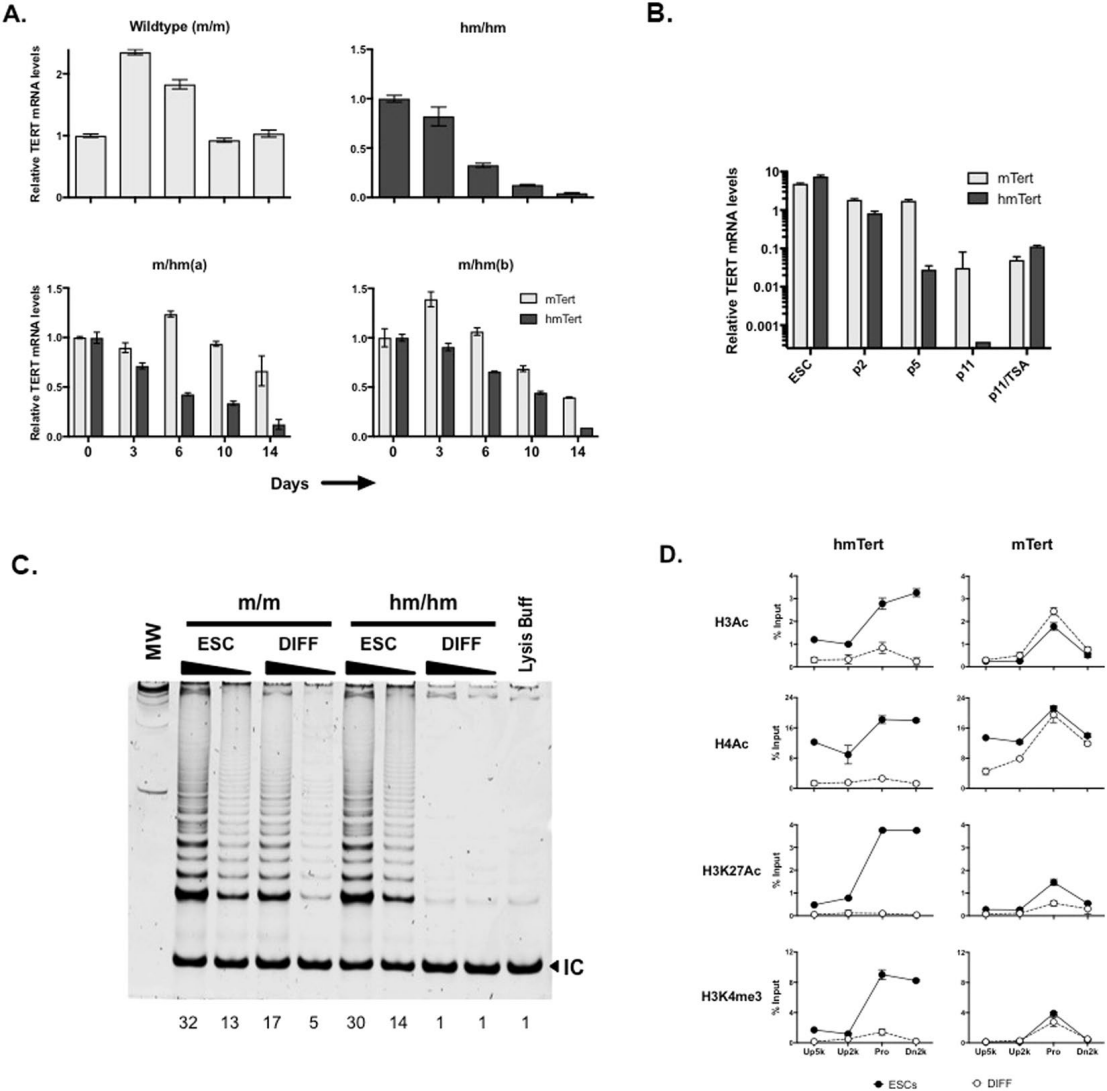
mRNA expression and telomerase activity from *mTert* and *hmTert* loci during *in vitro* ESC differentiation. (**A**) Tert mRNA expression in differentiating EB cultures. Tert mRNA were measured by RT-qPCR and normalized to 18S rRNA. Light gray, mTert; dark gray, hmTert. (**B**) mTert mRNA levels in differentiated fibroblast-like cells. 3-day old EBs (*m*/*hm*) were passaged in DMEM with 10% fetal bovine serum for 2, 5, and 11 times. Passage 11 cells were also treated for 24 h with 200 ng/ml TSA. mRNAs from *mTert* and *hmTert* loci were measured using specific primers spanning intron 2. (**C**) Telomerase activities in ESCs and differentiated cells. Telomerase activities in extracts of 2000 or 20 ESCs and fibroblast-like cells (passage 11) were measured using TRAP assay. Numbers below are relative intensities of all telomere bands in each lane, normalized to lysis buffer control (1). (**D**) Covalent histone modifications at genomic regions around the hmTert and mTert promoters in ESCs and differentiated cells. Chromatin fragments from ESCs (*m*/*hm*) and differentiated cells (DIFF) were precipitated using antibodies against specific histone marks, followed by qPCR analysis. H3Ac and H4Ac refer to actylated histones H3 and H4. H3K27Ac and H3K4me3 are acetylated K27 and trimethylated K4 residues of histone H3, respectively. Pro, hmTert or mTert promoters. Up5k, Up2k, and Dn2k are chromosomal sites 5-kb and 2-kb upstream and 2-kb downstream of the Tert promoters, respectively.

To determine the regulation of *hmTert* gene, Tert mRNA in differentiating EBs derived from ESCs with *hmTert* alleles were analyzed by RT-qPCR. As shown in Fig. 5A, the Tert mRNA levels were dramatically down-regulated during EB differentiation for 2 weeks in *hm*/*hm* cells, but not in wildtype *m*/*m* cells. Similarly in three clones of heterozygous *m*/*hm* cells, mRNA from the *hmTert* alleles decreased 8 and 15 folds as differentiation marker α-fetoprotein (AFP) increased during the differentiation process, whereas the down-regulation of expression from the *mTert* alleles was much less than the *hmTert* alleles in the same cells (Figs 5A and S3). Fibroblast-like cells from EBs of an *m*/*hm* clone were collected and passaged (Fig. S4). mRNA from both *mTert* and *hmTert* alleles progressively decreased upon passaging (Fig. 5B). The presence of incompletely differentiated cells might have contributed to the higher Tert mRNA levels in early passages of the cultures because fibroblast markers, type I collagen (Col1a1) and vimentin, progressively increased during passaging (Fig. S5). However, mRNA from the *hmTert* gene decreased much faster than that of *mTert*. The hmTert mRNA was about 100-fold lower than mTert mRNA after 11 passages. TSA treatment markedly induced hmTert mRNA, but had little effect on the level of mTert mRNA. Furthermore, telomerase activities were measured in ESCs and differentiated derivatives by TRAP assay. Whereas fibroblast-like cells derived from *m*/*m* ESCs contained a lower level of telomerase activity compared to that in undifferentiated ESCs, it was not detected in differentiated *hm*/*hm* cells (Fig. 5C), mimicking telomerase expression in human fibroblasts.

The epigenetic status of the *hmTert* locus in ESCs and their differentiated derivatives was also examined using chromatin immunoprecipitation. As shown in Fig. 5D, the region around the hmTert promoter was hyperacetylated at both histones H3 and H4 in ESCs and became deacetylated in differentiated cells. In contrast, the acetylation of histones at the *mTert* locus was not dramatically altered at the wildtype *mTert* locus during differentiation. As a control, the level of H3K4me3, a marker of active transcription, decreased significantly at the *hmTert*, but not *mTert*, locus. Altogether, these results indicated that the expression from the *hmTert* locus was tightly regulated and repressed by an HDAC-dependent mechanism in differentiated mouse cells, recapitulating the *hTERT* locus in human fibroblasts.

### Alternative splicing of hmTERT mRNAs

**Figure 6:**
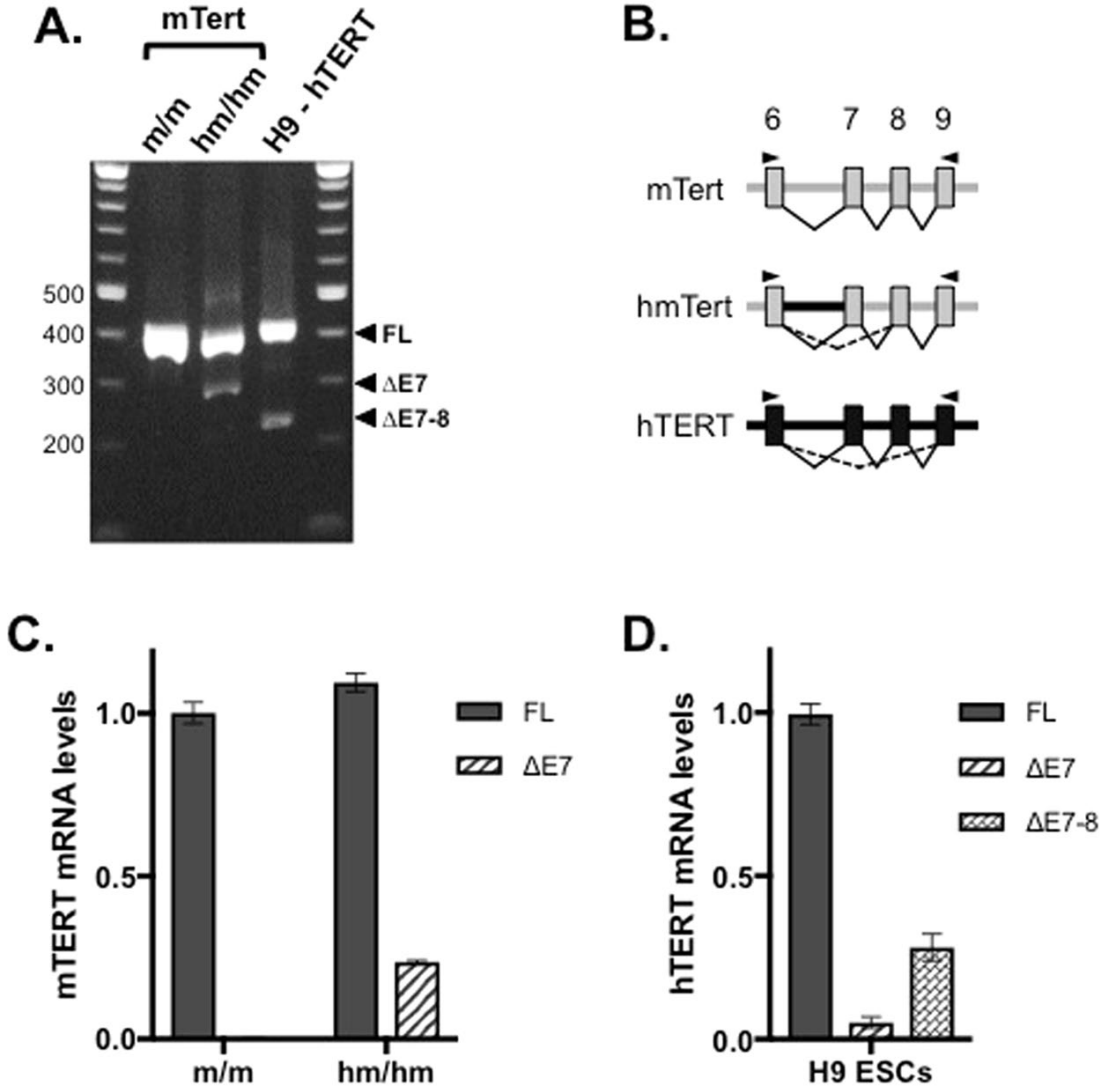
The impact of human intron 6 on hmTert splicing. (**A**) PCR analysis of mTert, hmTert, and hTERT mRNAs. Total RNAs from mouse and human ESCs were subjected to PCR analysis using primers spanning introns 6–8 and analyzed on an Agarose gel. Numbers on the left are sizes of molecular weight markers in bp. (**B**) Diagrams of the genomic regions spanning exons 6–9. Mouse exons and introns are designated by grey lines and boxes and human sequences are shown as black lines and boxes. Splicing patterns are indicated by thin lines below. Arrowheads show the positions of PCR primers used in panel (A,C). Alternative splicing of exon 7 at the *mTert* and *hmTert* loci. mTert mRNA levels in ESCs were determined by RT-qPCR analysis using primers spanning intron 6 (FL) or introns 6–7 (∆E7). (**D**) Splicing of exon 7 of the *hTERT* locus. hTERT mRNA levels in human H9 ESCs were measured by RT-qPCR analysis using primers spanning intron 6 (FL), introns 6–7 (∆E7), or introns 6–8 (∆E7-8).

The transcript from the *hTERT* gene is spliced into several forms, providing an additional regulation for its activity^7^. The major alternative splicing product is the minus beta form, which skips exons 7–8 and likely encodes an inactive form due to a downstream reading frame shift. It was reported that genomic sequence within human intron 6 was essential for this splicing event^18^. Thus, we examined splicing patterns around human I6 sequence in the *hmTert* locus by RT-PCR analysis using primers spanning exons 6 to 9. As shown in Fig. 6, whereas the *mTert* gene produced the full-length (FL) mRNA exclusively, the *hmTert* locus yielded an additional short form consistent with the skipping of exon 7 (∆E7), which was validated by sequencing the PCR fragment. To measure the levels of splicing variants quantitatively, RT-qPCR analysis was performed using primers specific for FL and ∆E7. The levels of FL form were similar in *m*/*m* and *hm*/*hm* ESCs, but *hm*/*hm* cells contained an additional 1/5 amount of ∆E7 form (Fig. 6C). Similarly, the endogenous *hTERT* gene in human ESCs also produced mostly the FL form, one quarter of ∆E7-8, and little ∆E7 (Fig. 6D). In addition, the ∆E7 mRNA was also induced by TSA treatment in differentiated cells (Fig. S6). Therefore, human I6 sequence facilitated the splicing of ∆E7-8 in the *hTERT* gene and ∆E7 in the *hmTert* locus in human and mouse ESCs, respectively. Although it was unlikely that this alternative splicing affected telomerase activity significantly in ESCs, the presence of I6 in the *hmTert* gene might help it to recapitulate the regulation of *hTERT* gene in somatic cells.

## Discussion

A principal difference between mice and humans relates to a longtime observation that murine fibroblasts growing in culture undergo spontaneous immortalization at a high frequency, owing to their long telomeres and constitutive telomerase expression^19^. Data from multiple laboratories, including our own, have indicated that transcriptional regulation of the *TERT* genes is fundamental to this interspecies difference^3,8,20^. As we have shown in our previous studies^10,21^, a central difference between the regulation of *hTERT* and *mTert* genes is the stringent repression of hTERT promoter in somatic cells. In this report, we identified 5IR, I2, & I6 as genomic sequences that confer human-specific repression during differentiation. The chromatinized BAC reporter, M(h5IRI2I6), containing human 5IR, I2, & I6 replacing their mouse counterparts in M(wt), was much more active in Tel^+^ human fibroblasts than in Tel^−^ cells, recapitulating the endogenous *hTERT* gene in host cells. This reporter was active in ESCs and repressed upon differentiation; hence, we knocked this entire 47-kb chimeric genomic sequence (from 5IR to I6) into the *mTert* locus in ESCs. The resulting ESC clone with homozygous *hmTert* loci contained a high level of telomerase and was able to proliferate for over 400 population doublings without detectable telomere deficiencies, indicating that the Tert protein expressed from this engineered gene was fully functional. Moreover, the regulation of *hmTert* gene recapitulated that of the *hTERT* gene during *in vitro* ESC differentiation.

Our ultimate goal is to engineer a mouse strain with humanized telomere homeostasis. Mouse models of human diseases have become a central part of biomedical research. Laboratory mice provide the most experimentally accessible mammalian models that bear close similarities of genes, organs, and systemic physiology with humans. However, species-specific telomerase regulation and telomere homeostasis remain to be a major challenge in using mouse models to study human diseases. This is because telomere homeostasis is involved in the pathogenic processes of many types of diseases, especially those related to aging and cancer. One approach of generating such a mouse model is to complement *mTert* −/− mice with a transgenic *hTERT* gene. However, such an approach is not possible because the hTERT protein does not function with mTerc RNA^22,23^. In contrast, the Tert protein encoded by the *hmTert* locus is identical to that of the wildtype *mTert* gene. Thus, humanization of the *mTert* locus, like the *hmTert* allele we engineered here, is a new and practical approach for the generation of such a mouse model.

It remains to be elucidated how distal elements within the human 5IR, I2, and I6 regions control the regulation of *hTERT* gene. It has been noted that these human genomic regions contain abundant repetitive sequences, much more than their mouse counterparts. These repetitive elements include Alu, LINE, endogenous retroviral elements, and variable number tandem repeats (VNTRs). The functions of these elements in regulating hTERT expression are a focus of ongoing studies. For example, I6 was found to be involved in alternative splicing of the hTERT transcript^18^. In particular, a 1-kb VNTR within this intron was able to alter hTERT mRNA splicing^24^, and it also affected mRNA splicing in the *hmTert* locus (Fig. 6). The presence of abundant repetitive elements at the *hTERT* locus also likely helps to establish a repressive chromatin environment and repress its promoter activity.

In short, the new *hmTert* locus encoded an mTert protein that was able to maintain telomere length during long-term ESC proliferation. Its expression was repressed in differentiated cells by a HDAC-dependent mechanism, similar to the native *hTERT* gene. This *hmTert* locus provided a foundation for our long-term goal of generating a novel mouse model with humanized telomere homeostasis.

## Materials and Methods

### Bacterial Artificial Chromosomes (BACs) and Plasmids

BAC reporters H(wt), M(wt), H(mPro) and M(hPro), were described previously^11,12,17^. Other chimeric BACs and the targeting BAC were constructed using a multi-step BAC recombineering strategy^25,26^. Briefly, 5′ intergenic (h5IR, 22-kb), introns 2 (hI2, 10-kb) and 6 (hI6, 5.5-kb) of the *hTERT* gene replaced the syntenic regions in M(wt) to generate M(h5IR), M(h5IRI2), and M(h5IRI2I6), respectively (Fig. 1). The targeting BAC for the knock-in experiment was constructed using M(h5IRI2I6) with the following modifications: (1) The *Rluc* cassette was removed and the mTert initiation codon was restored; (2) Nine silent nucleotide substitutions were introduced into the coding region near 3′ end of exon 2 (AAAGTAGAGGATTGCCACTGGCTCCGCAGCAGCCCGG to AA*G*GT*C*GA*A*GA*C*TGCCACTGGCT*GA*G*GTC*CAGCCCGG); (3) A puromycin-resistant marker (Puro), surrounded by a pair of *lox*511 sites, was inserted behind the 3′ end of CRR9 gene; (4) A thymidine kinase (TK) cassette replaced exons 1–12 of the *CRR9* gene; (5) A 300-bp sequence from the 5′ end of mTert intron 2 was inserted before the hTERT intron 2 to restore its efficient splicing; (6) Sequences downstream of mTert exon 11 were deleted. The resulting BAC had about 4- & 3-kb of 5′ and 3′ homology arms, respectively. Single guide RNAs (sgRNAs) targeting mouse introns 2 and 6 were cloned into pX458^27^.

### Cell culture and ESCs differentiation

Telomerase-positive 3C167b3.1 (Tel^+^) and -negative GM847.7 (Tel^−^) human fibroblast lines and mouse ESCs Tc1 (129S6/SvEvTac) were cultured as previously described^11,12^. BAC reporters were integrated into Tel^+^, Tel^−^, and T2-5 (a clone of Tc1 cells), all containing a single BAC acceptor site, using RMBT^12,17^. ESC differentiation into embryoid bodies (EBs) and fibroblast-like cells were also described^28^. Individual EBs were collected for luciferase assay. 3d-old EBs were treated with 10^−7^ M Retinoic Acid (RA) (Sigma-Aldrich, US) for 2d and cultured for another 2d without RA. Adherent cells migrated out of EBs were collected and passaged. Fibroblast-like cells were characterized by the expression of vimentin^11^.

### Genomic Knock-In

Knock-in experiments were performed by cotransfection of the targeting BAC and two pX458-sgRNAs (targeting the 5′ intergenic region and intron 6 of the *mTert* locus) into Tc1 cells^29^ (Fig. 3A). Following transfection, puromycin and GCV-resistant colonies are isolated. Genomic PCR and Southern analyses were performed to identify clones with correct knock-in in all three regions, 5IR, I2, and I6 (Fig. S1A,B)^8,10^. Probes are listed in Supplemental Table S1 and results are described in Table S2. Finally, the puromycin marker was removed following transient transfection of pCBM, a Cre-expressing plasmid^17^. As a control, ESCs with *mTert* homozygous knockout were also isolated following transfection of the abovementioned pX458-sgRNA plasmids.

### Gene expression analyses

Luciferase activities were measured using Dual-Luciferase Reporter Assay System (Promega, Madison, WI). *Firefly* luciferase *(Fluc)* from the *CRR9* genes was constitutively expressed in ESCs and differentiated cells. *Renilla* luciferase *(Rluc)* activities from *TERT* promoters were normalized to *Fluc* activity^17^ and the data were verified by normalizing *Rluc* activity to the number of cells, as determined by thiazolyl blue tetrazolium bromide (MTT) assay (data not shown). Quantitative RT-PCR (RT-qPCR) assays were conducted as previously described^11^.

### Telomerase activity and telomere length analyses

Telomerase activities were determined using a modified telomeric repeat amplification protocol (TRAP) assay^30,31^. Telomere length was measured by quantitative fluorescence *in situ* hybridization (qFISH). Metaphases were prepared and qFISH was performed as described previously^32,33^ using peptide nucleic acid (PNA) probes according to manufacturer’s instruction (PNA Bio Inc.). PNA probes were TelC-Cy3 (CCCTAA repeats) for telomere and CENPB-FAM (ATTCGTTGGAAACGGGA) for centromere. Fluorescence images were captured using Axioscope Z2 (Zeiss, Germany). The fluorescence signals were integrated from at least 15 metaphases. Quantitative image analyses of telomere were done following the procedure as described by Perner *et al*.^34^ with Isis software (MetaSystems, Altlussheim, Germany).

Telomere length in mESCs was also assessed by telomeric restriction fragment analysis. Genomic DNAs were digested with Hinf I and Rsa I, and subjected to pulsed-field gel electrophoresis using CHEF-DR III Pulsed field Electrophoresis Systems (Bio-Rad), followed by Southern blotting. Telomere restriction fragments were hybridized to a (TTAGGG)_3_-Biotin probe and detected North2South™ Chemiluminescent Hybridization and Detection Kit (Thermo Scientific).

### Statistical analysis

Two tails Student’s t test was used to compare relative gene expression by RT-qPCR.

The datasets generated during and/or analysed during the current study are available from the corresponding author on reasonable request.

## Supplementary information

Supplementary Information
