# Author Correction: Engineering a humanized telomerase reverse transcriptase gene in mouse embryonic stem cells

**DOI:** 10.1038/s41598-020-58142-z

**Published:** 2020-01-21

**Authors:** De Cheng, Yuanjun Zhao, Fan Zhang, Jinglong Zhang, Shuwen Wang, Jiyue Zhu

**Affiliations:** 1 Department of Pharmaceutical Sciences, Washington State University College of Pharmacy and Pharmaceutical Sciences, Spokane, Washington USA; 2 Department of C & M Physiology, Pennsylvania State University College of Medicine, Hershey, Pennsylvania USA

Correction to: *Scientific Reports* 10.1038/s41598-019-46160-5, published online 04 July 2019

This Article contains errors in Figure 4D, where the incorrect image is shown for passage 60 of mTert/mTert embryonic stem cells. The correct Figure appears below as Figure 1.

**Figure 1:**
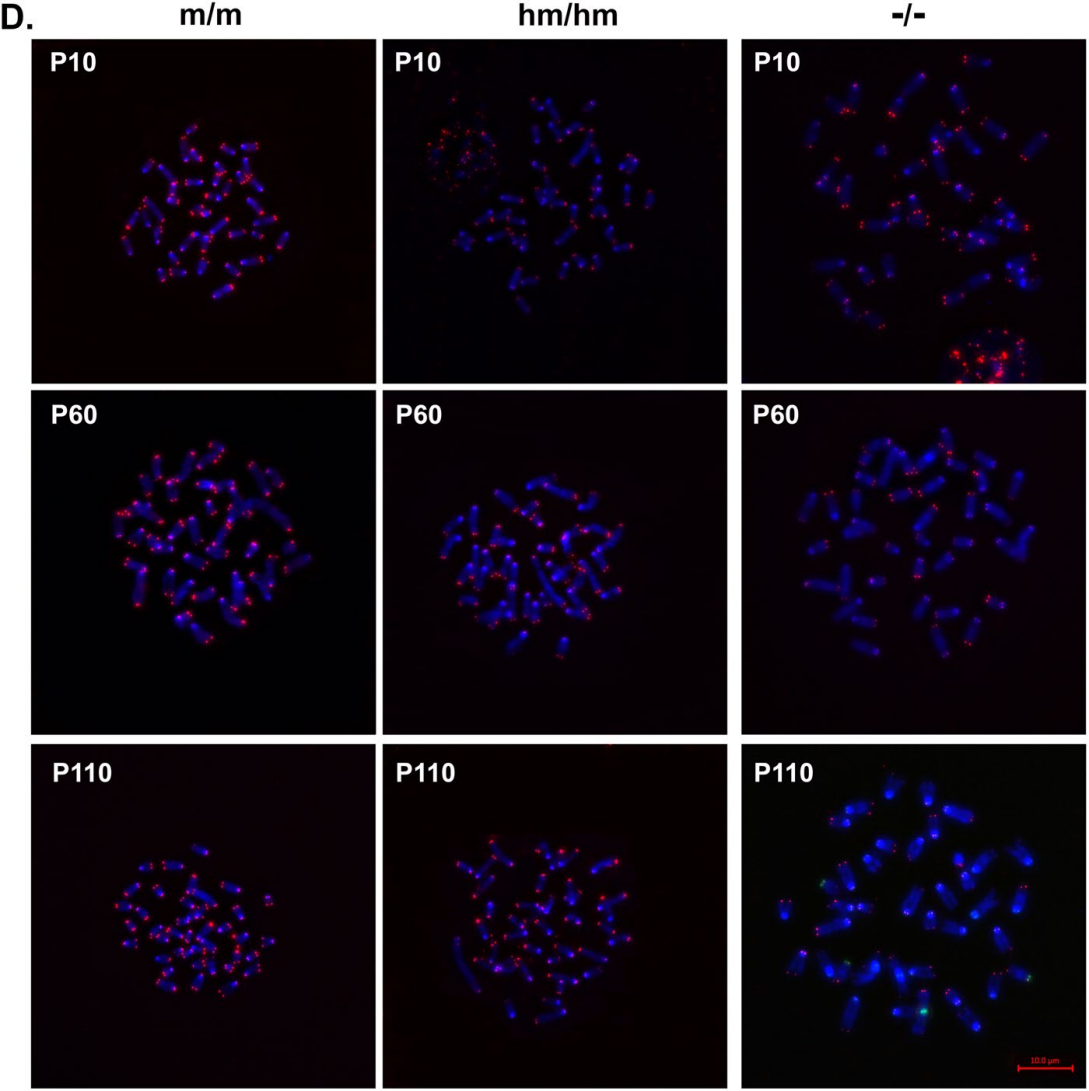
.

The conclusions of the Article are unaffected by these changes.

