# Supplementary material for: Engineering a humanized telomerase reverse transcriptase gene in mouse embryonic stem cells
Source: Sci Rep. 2019 Jul 4;9:9683. doi: 10.1038/s41598-019-46160-5 (PMC6609615; doi:10.1038/s41598-019-46160-5)
Supplement: Supplementary file 1 — Supplementary Information [file 41598_2019_46160_MOESM1_ESM.pdf]

Supporting Information for

Engineering a humanized telomerase reverse transcriptase gene in mouse embryonic stem cells

De Cheng, Yuanjun Zhao, Fan Zhang, Jinglong Zhang, Shuwen Wang, and Jiyue Zhu

Figure S1

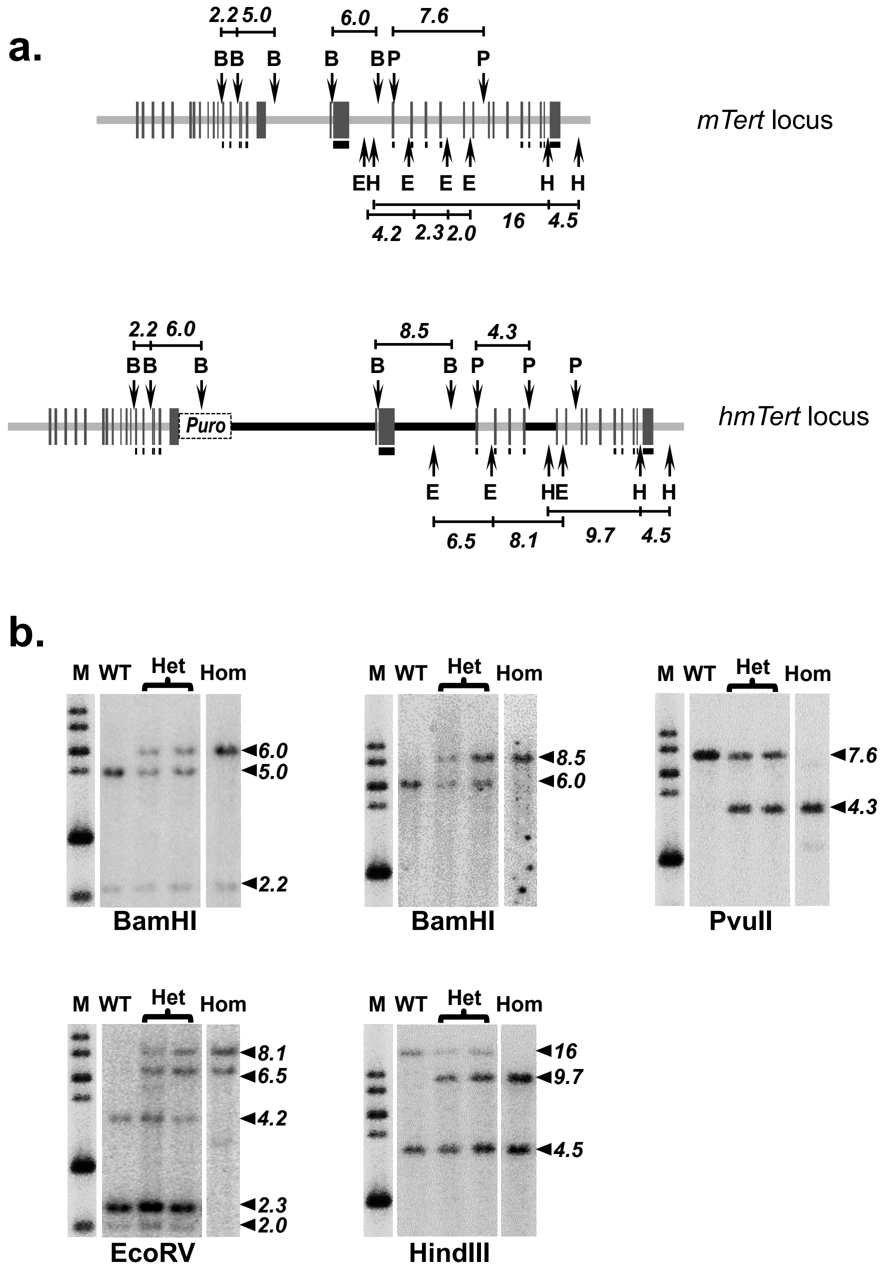

Figure S1. Characterization of the *hmTert* locus. **a**. Genomic maps of the *mTert* and *hmTert* loci. Numbers indicate sizes of restriction fragments in kilobases. B, BamHI; P, PvuII; E, EcoRV; & H, HindIII. Small bars underneath the genomic maps are positions of cDNA probes. **b**. Southern blot analyses of the *mTert* and *hmTert* loci. Genomic DNAs were digested with restriction enzymes, as indicated below each panel, and analyzed on 0.7% agarose gels, and hybridized to probes shown in **a**. WT, *mTert*/*mTert*; Het, *mTert*/*hmTert*; Hom, *hmTert*/*hmTert*; M, DNA size marker.

Figure S2

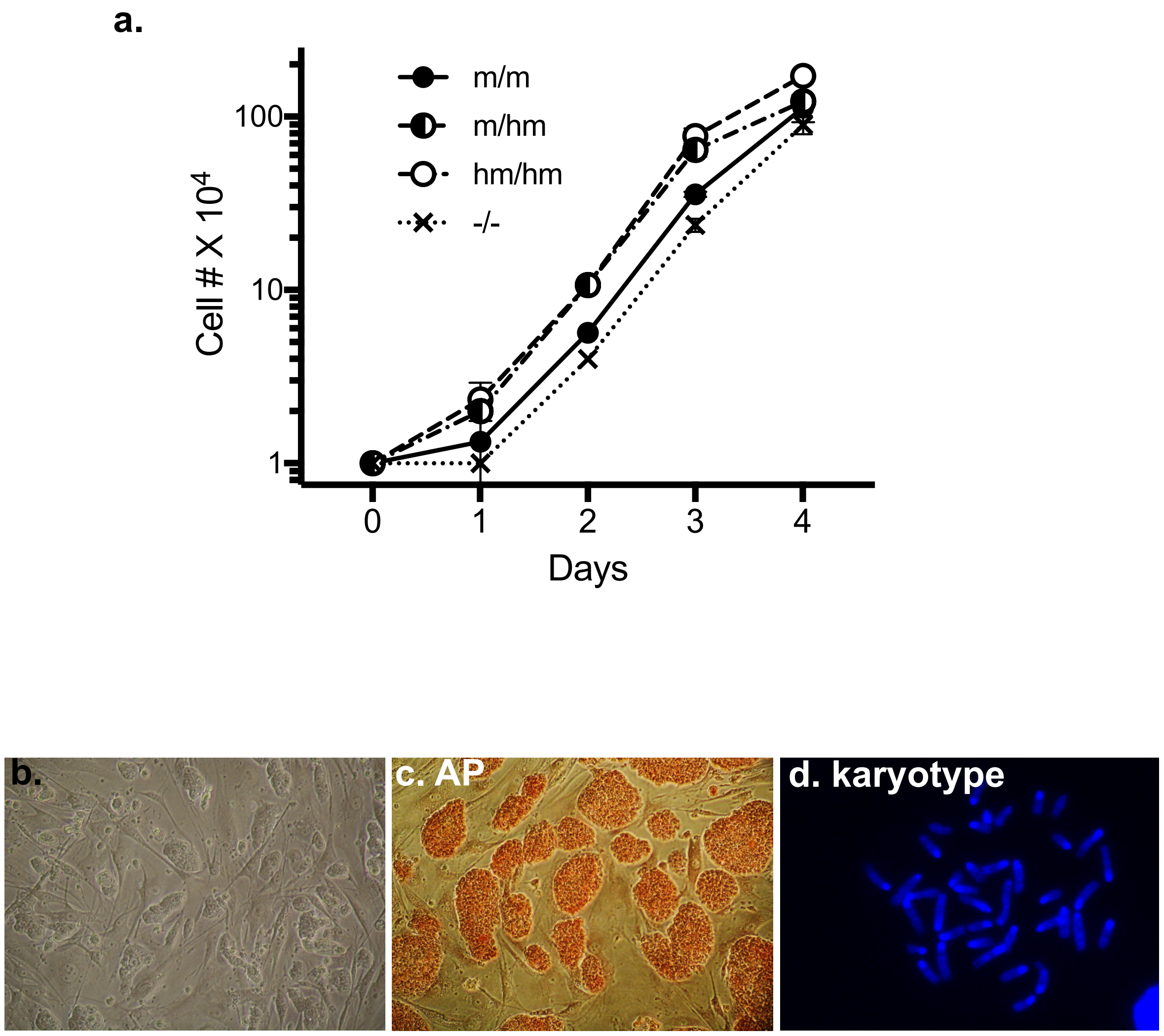

Figure S2. ESCs with *hmTert* alleles. **a**. ESC proliferation. **b**. Images of ESC colonies (hm/hm). **c**. ESCs (hm/hm) stained with alkaline phosphatase (AP). **d**. Karyotype of a ESC (hm/hm) with 40 chromosomes.

Figure S3

a.

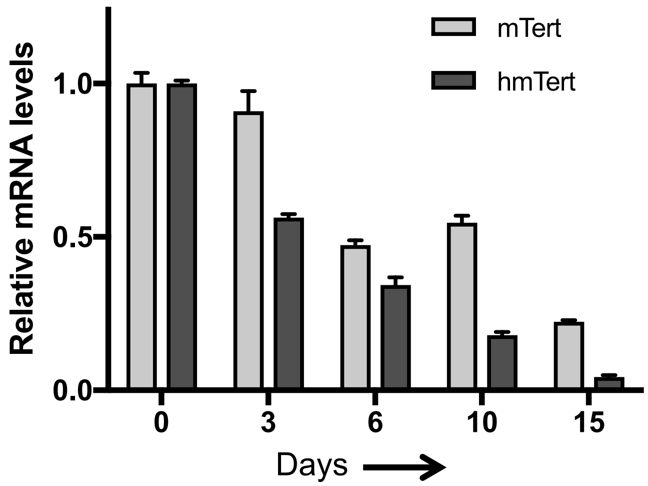
mTert
hmTert
Relative mRNA levels
1.0
0.5
0.0
0
3
6
10
15
Days

b.

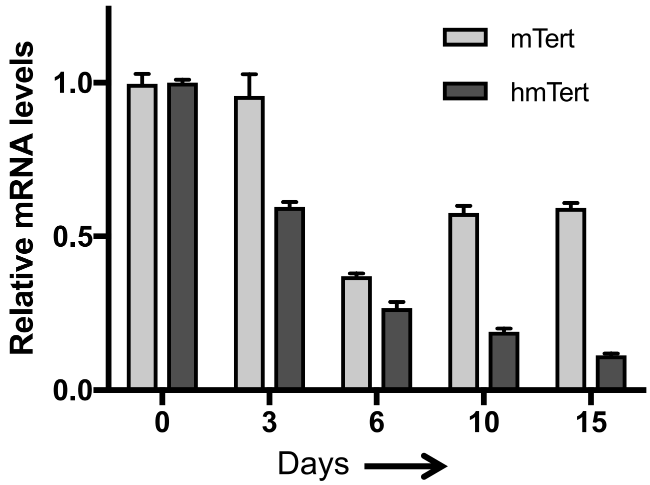
mTert
hmTert
Relative mRNA levels
1.0
0.5
0.0
0
3
6
10
15
Days

Figure S3

c.

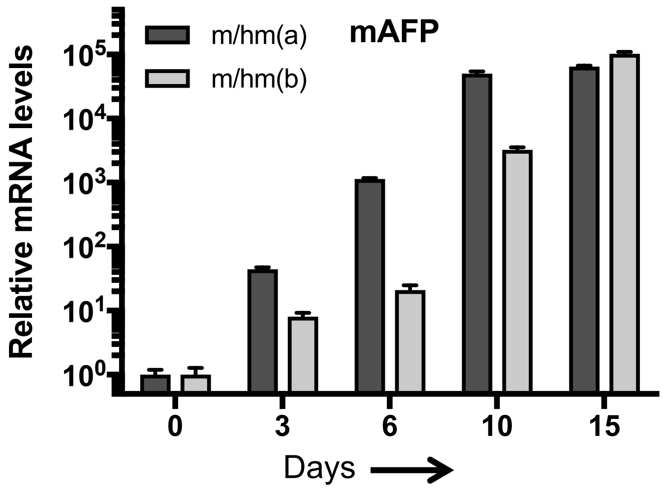

Figure S3. mRNA expression during *in vitro* ESC differentiation. The experiment was performed as described in Figure 5A. Tert mRNAs from differentiating EBs derived from ESC clone m/hm(c) were measured by RT-qPCR and normalized to 18S rRNA (**a**) or β-actin mRNA (**b**). **c.** AFP mRNA expression in differentiating ESC clones, m/hm(a) and m/hm(b), normalized to 18S rRNA.

Figure S4

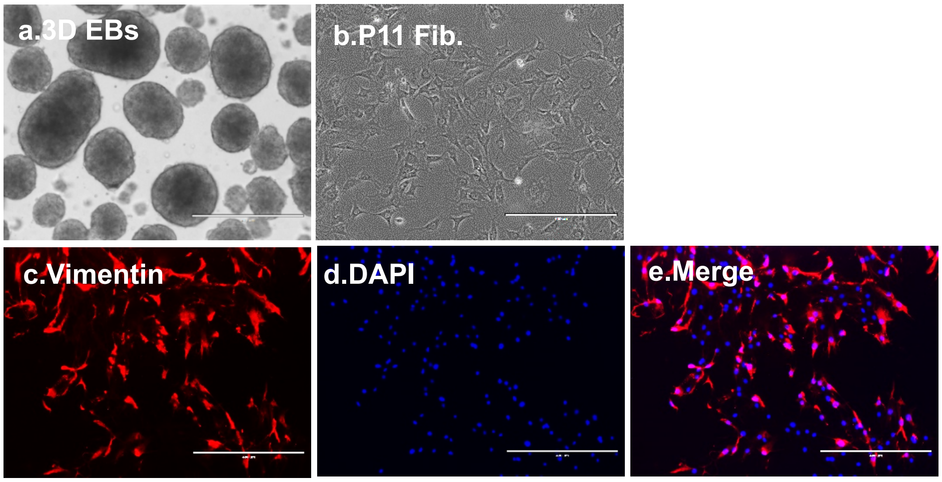

Figure S4. ESC differentiation. **a**. Differentiating EB cultures. **b**. Images of differentiated fibroblast-like cells (passage 11). **c-e**. Expression of vimentin in fibroblast-like cells. Differentiated cells were stained with an anti-vimentin antibody: b, bright field; c, vimentin antibody staining; d, DAPI staining; e, merged image.

Figure S5

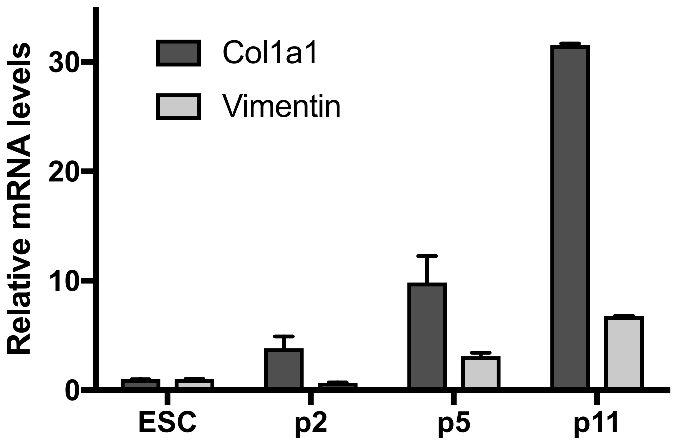

Figure S5. Col1a1 and vimentin mRNA expression in differentiating fibroblast-like cultures. The mRNA levels were normalized to 18SrRNA.

Figure S6

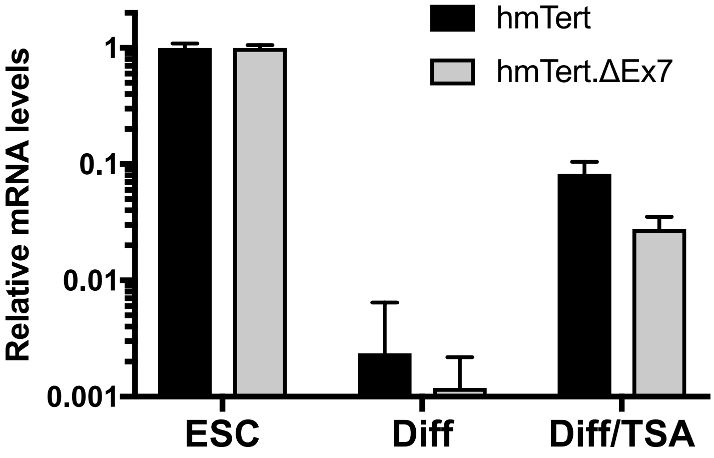

Figure S6. hmTert mRNA splicing upon TSA treatment. hmTert mRNA and its variant without exon 7 were measured as described in Figure 6C. Differentiated Fibroblast-like cells were treated for 24h with 200ng/ml TSA. mRNA levels were normalized 18SrRNA. The levels shown in differentiated cells are relative to their respective levels in undifferentiated ESCs.

Table S1
BAC knock-In experiment in ESCs

Table S2a. Identification of ESCs with homogenous recombination

	Puromycin positive	Puro+ & GCV+	Recombination at 5' Homologous Arm
Methods of identification	Selection	Selection	PCR
Total # colonies	800	110	84
Percentages	-	13.75%	10.50%

Table S2b. Summary of Southern Blotting Experiment

Genotypes	5'IR/mTert+	5'IR+I2/mTert+	hmTert/mTert+	hmTert/mTert-	hmTert/hmTert
Number of clones	3	3	9	9	1

Total number of clones examined: 42

5'IR: Knock-in of only the 5' intergenic region of the hTERT gene
5'IR+I2: Knock-in of the 5' intergenic region and intron 2 of the hTERT gene
hmTert: Knock-in of the 5' intergenic region, introns 2 and 6 of the hTERT gene
mTert-: Genomic deletion from 5' intergenic region to intron 6 of the mTert locus

Table S2
Probes for Southern blots

Probe	Probe region	Probe size (bp)	DNA fragments
Probe A	mCRR9 cDNA Exon11-Exon17	738	KpnI/NcoI
Probe B	mTERT cDNA Exon2-Exon3	1037	BamHI/EcoRV
Probe C	mTERT cDNA Exon3-Exon6	673	BamHI/BstEII
Probe D	mTERT cDNA Exon3-Exon7	744	PCR (mTERT cDNA nt 1636-2380)
Probe E	mTERT cDNA Exon12-Exon16	756	PCR (mTERT cDNA nt 2847-3602)

sgRNA sequences

sgRNA1	5'-AAGGATGAGGTTGGGCCAAT	targeting the 5' interginic region of the mTert gene
sgRNA2	5'-TCTGCAATGGCGTGGTCCCA	targeting intron 6 of the mTert gene

Table S3. Lists of PCR primers

Genomic DNA PCR primers

Targets	Forward, 5'-->3'	Reverse, 5'-->3'	Amplicon Sizes (bp)
5' end of homologous recombination	ATTCTCGGGACCCTGTGC	GCGGGACTATGGTGGCTG	4888
TK region in Targeting BAC	CGCCTTCTATGAAAGGTTGG	AGACTCACGTGGGGAGACAC	467
hTERT Promoter	GCGGCGCGAGTTTCAG	AAGCAGCTCAAAGCCAAAAG	485
hTERT Intron 2	TGTGGAAGATGAAGGTCGAA	GTTCTCGTCCCCACCTCT	427
hTERT Intron 6	ATCTCCTGCAGGTTTGCTGT	AGAAACGCATCACAGACACG	363
Puromycin resistance marker	CGCCCTCTTCTGGTGTATC	AGGCAAGAAGATTGTGTGAAC	333
human 5' intergenic region	CCAAAGGCGTAAAACAGGAA	CCTCGTGTACTTTCCCTTGC	140
Mouse 5' intergenic region	GGGGGCTCTAGGAAACATTC	AAGAGGAAAGGATGAGGTTGG	98

qRT-PCR Primers

Targets	Forward, 5'-->3'	Reverse, 5'-->3'	Amplicon Sizes (bp)
Humanized mTERT mRNA Mut	TGTGGAAGATGAAGGTCGAA	CCACGTATGTGTCCATCAGC	135
Endogenous mTERT mRNA	TGAAAGTAGAGGATTGCCACTG	CCACGTATGTGTCCATCAGC	126
18s RNA intenal control	TAGAGGGACAAGTGGCGTTC	CGCTGAGCCAGTCAGTGT	104
qRT.mTERT.1F/R	TTCTAGACTTGCAGGTGAACAGCC	TTCCTAACACGCTGGTCAAAGGGA	124
Full length mTERT mRNA	CTGAGGAACTCCGTTGTCATC	TAGCACCTGTCACCAATCTTTAC	119
mTERT mRNA ΔExon7	CACAAGTCCTTTAGGAGACAGAG	TAGCACCTGTCACCAATCTTTAC	113
Full length hTERT mRNA	CGTCGTCATCGAGCAGAG	GCACTGGACGTAGGACTTG	118
hTERT mRNA ΔExon7	AGGCCTTCAAGAGCCACAG	GCACTGGACGTAGGACTTG	119
hTERT mRNA ΔExon7-8	GCCTTCAAGAGCCACGTCC	CAAACAGCTTGTTCTCCATGTC	110
mβ-Actin	TTTCCAGCCTTCCTTCTTGG	GGCATAGAGGTCTTTACGGATG	103
mAFP	TTTCACAGAAGAGGGTCCAAAG	GGCTCACACCAAAGAGTCAA	113
mCol1a1	ACAAGGTGACAGAGGCATAAA	ACCAGGAGAACCAGGAGAA	79
mVimentin	CATTGAGATCGCCACCTACAG	TCTCTCAGGTTCAGGGAAGAA	93

RT-PCR Primers

Targets	Forward, 5'-->3'	Reverse, 5'-->3'	Amplicon Sizes (bp)
mTERT mRNA Splicing	AGGGTAAGCTGGTGGAGGTT	CAAACAGCTTGTTCTCCATGTC	408, 306, 220
hTERT mRNA Splicing	CATCGCCAGCATCATCAAAC	CAAGAAATCATCCACCAAACG	430, 334, 248

TRAP Assay oligos

TS	AATCCGTCGAGCAGAGTT
NT	ATCGCTTCTCGGCCTTTT
TSNT	AATCCGTCGAGCAGAGTTAAAAGGCCGAGAAGCGAT
ACX	GCGCGGCTTACCCTTACCCTTACCCTAACC
